# Prevalence and Predictors of Prolonged Cognitive and Psychological Symptoms Following COVID-19 in the United States

**DOI:** 10.3389/fnagi.2021.690383

**Published:** 2021-07-19

**Authors:** Jennifer A. Frontera, Ariane Lewis, Kara Melmed, Jessica Lin, Daniel Kondziella, Raimund Helbok, Shadi Yaghi, Sharon Meropol, Thomas Wisniewski, Laura Balcer, Steven L. Galetta

**Affiliations:** ^1^Department of Neurology, New York University Grossman School of Medicine, New York, NY, United States; ^2^Rigshospitalet, Department of Neurology, Copenhagen University Hospital, Copenhagen, Denmark; ^3^Department of Clinical Medicine, University of Copenhagen, Copenhagen, Denmark; ^4^Department of Neurology, University of Innsbruck, Innsbruck, Austria; ^5^Department of Neurology, School of Medicine, Brown University, Providence, RI, United States

**Keywords:** COVID-19, long-hauler, cognitive, stressors, Community Dwellers, post-acute sequelae of SARS-CoV-2 infection

## Abstract

**Background/Objectives:**

Little is known regarding the prevalence and predictors of prolonged cognitive and psychological symptoms of COVID-19 among community-dwellers. We aimed to quantitatively measure self-reported metrics of fatigue, cognitive dysfunction, anxiety, depression, and sleep and identify factors associated with these metrics among United States residents with or without COVID-19.

**Methods:**

We solicited 1000 adult United States residents for an online survey conducted February 3–5, 2021 utilizing a commercial crowdsourcing community research platform. The platform curates eligible participants to approximate United States demographics by age, sex, and race proportions. COVID-19 was diagnosed by laboratory testing and/or by exposure to a known positive contact with subsequent typical symptoms. Prolonged COVID-19 was self-reported and coded for those with symptoms ≥ 1 month following initial diagnosis. The primary outcomes were NIH PROMIS/Neuro-QoL short-form T-scores for fatigue, cognitive dysfunction, anxiety, depression, and sleep compared among those with prolonged COVID-19 symptoms, COVID-19 without prolonged symptoms and COVID-19 negative subjects. Multivariable backwards step-wise logistic regression models were constructed to predict abnormal Neuro-QoL metrics.

**Results:**

Among 999 respondents, the average age was 45 years (range 18–84), 49% were male, 76 (7.6%) had a history of COVID-19 and 19/76 (25%) COVID-19 positive participants reported prolonged symptoms lasting a median of 4 months (range 1–13). Prolonged COVID-19 participants were more often younger, female, Hispanic, and had a history of depression/mood/thought disorder (all *P* < 0.05). They experienced significantly higher rates of unemployment and financial insecurity, and their symptoms created greater interference with work and household activities compared to other COVID-19 status groups (all *P* < 0.05). After adjusting for demographics, past medical history and stressor covariates in multivariable logistic regression analysis, COVID-19 status was independently predictive of worse Neuro-QoL cognitive dysfunction scores (adjusted OR 11.52, 95% CI 1.01–2.28, *P* = 0.047), but there were no significant differences in quantitative measures of anxiety, depression, fatigue, or sleep.

**Conclusion:**

Prolonged symptoms occurred in 25% of COVID-19 positive participants, and NeuroQoL cognitive dysfunction scores were significantly worse among COVID-19 positive subjects, even after accounting for demographic and stressor covariates. Fatigue, anxiety, depression, and sleep scores did not differ between COVID-19 positive and negative respondents.

## Introduction

Prolonged symptoms of COVID-19 including fatigue, cognitive abnormalities and mood disorders have been reported (Carfi et al., 2020; Tenforde et al., 2020; Al-Aly et al., 2021; Huang et al., 2021; Nalbandian et al., 2021), however, the prevalence of these symptoms in the general population is not known. Furthermore, community members without COVID-19 may suffer similar symptoms related to social and economic stressors encountered during the global pandemic. Qualitative reports of COVID-19-related symptoms are limited in their ability to assess the severity of physical and psychological manifestations. Self-reported health status batteries that have been validated in clinical and reference populations provide quantitative measures of symptoms and may help parse the impact of SARS-CoV-2 infection from pandemic-related stressors.

We aimed to estimate the prevalence of symptoms of anxiety, depression, fatigue, sleep abnormalities, and subjective cognitive dysfunction among United States residents with or without the diagnosis of COVID-19 using quantitative NIH PROMIS/Neuro-QoL metrics.

## Materials and Methods

### Design and Participants

We surveyed an unprimed sample of adult (≥18 years old) community-dwelling United States residents between February 3–5, 2021, utilizing the online platform Prolific.co^1^. Prolific.co, which is compliant with European Union General Data Protection Regulations (GDPR), is a crowdsourcing platform developed to recruit human subjects for research purposes (Kondziella et al., 2020). Data quality, participant diversity and honesty of responses compare favorably with other similar crowdsourcing/micro-jobbing platforms (Peer et al., 2017). Prolific has approximately 148,000 participants representing all 50 US states that routinely participate in community research surveys. The site has security checks to ensure that bots are not infiltrating the site. Potential survey participants were presented with a generic survey title (“Prevalence of Medical Conditions among Community Dwellers”) to avoid influencing survey participation. Survey questions specifically pertaining to COVID-19 status and prolonged COVID-19 symptoms or “long-hauler” syndrome were placed at the end of the survey to avoid confounding of responses to Neuro-QoL metrics (Supplementary Table 1). All Neuro-QoL batteries inquired about self-reported health within the “past 7 days”. A representative respondent sample reflecting age, sex and race proportions in the United States population was automatically curated by the survey platform utilizing United States Census Bureau data with a target of 1,000 responses (Table 1). Regions of the United States were defined according to United States Census Bureau standards (Northeast, Midwest, South, and West) (U.S. Census Bureau Map of the United States Showing Census Divisions and Regions, 2021), and population centers were characterized as rural, suburban, or urban.

**TABLE 1:** Demographics of survey respondents (*N* = 999) compared to United States census data from 2020*.

	Survey respondents	United States census data
Age (years) – median, 95% CI	45(95%*CI*44−46)	39
Sex (male),%, 95% CI	490/999(49%,95%*CI*46−52%)	162,478,564/328,239,523 (49.5%)
**Race**		
White, *N* (%, 95% CI)	765/999(77%,95%*CI*74−79%)	236,332,457/328,239,523 (72%)
Black, *N* (%, 95% CI)	130/999(13%,95%*CI*11−15%)	42,014,659/328,239,523 (12.8%)
Asian, *N* (%, 95% CI)	68/999(7%,95%*CI*5−9%)	18,709,653/328,239,523 (5.7%)
American Indian and Alaska Native, *N* (%, 95% CI)	4/999(0.4%,95%*CI*0.1−1%)	29,541,557/328,239,523 (0.9%)
Native Hawaiian/Pacific Islander, *N* (%, 95% CI)	2/999(0.2%,95%*CI*0.02−0.7%)	656,479/328,239,523 (0.2%)
Other or mixed race, *N* (%, 95% CI)	26/999(3%,95%*CI*2−4%)	27,572,120/328,239,523 (8.4%)
Hispanic ethnicity, *N* (%, 95% CI)	47/999(5%,95%*CI*4−6%)	60,396,072/328,239,523 (18.4%)
**Population by United States Regions**		
Northeast, *N* (%, 95% CI)	197/999(20%,95%*CI*17−22%)	17.1%, 95% CI
Midwest, *N* (%, 95% CI)	209/999(21%,95%*CI*18−24%)	20.8%, 95% CI
South, *N* (%, 95% CI)	413/999(41%,95%*CI*38−44%)	38.3%, 95% CI
West, *N* (%, 95% CI)	180/999(18%,95%*CI*16−21%)	23.9%, 95% CI

*https://data.census.gov/cedsci/profile?q=United%20States&g=0100000US, accessed February 6, 2021.

### Exposure

A diagnosis of COVID-19 was coded for subjects that had self-reported positive SARS-CoV-2 RT-PCR or antibody testing or for those that had exposure to a person with SARS-CoV-2 infection *and* subsequent symptoms of COVID-19 including fever > 99.5F, new onset cough, shortness of breath, muscle pain, headache, sore throat and/or loss of taste/smell (Centers for Disease Control and Prevention Covid-19, 2019). “Prolonged COVID-19” was self-reported among COVID-19 participants who continued to have symptoms ≥1 month after initial diagnosis (Datta et al., 2020; Lerner et al., 2021). COVID-19 status was trichotomized as negative, positive without prolonged symptoms and positive *with* prolonged symptoms. Symptom lists were developed from Centers for Disease Control and Prevention (CDC) (CDC, 2019; Centers for Disease Control and Prevention Covid-19, 2019) and World Health Organization (WHO) post-COVID questionnaires (World Health Organization, 2021).

### Outcome Measures

The primary outcomes were the NIH/NINDS PROMIS Quality of Life in Neurological Disorders (Neuro-QoL, 0000; Cella et al., 2012; Gershon et al., 2012; NIH, 2015) (NeuroQoL) short form self-reported health measures of anxiety, depression, fatigue, cognition and sleep. Neuro-QoL raw scores were converted into T-scores with a mean of 50 and standard deviation of 10 in a reference population (United States general population or clinical sample) (Neuro-QoL, 2021). Higher T-scores indicate worse self-reported health for the anxiety, depression, fatigue, and sleep metrics, while lower scores indicate worse self-reported health for the cognitive function metric.

### Statistical Analyses

This study was powered to detect a T-score mean difference of five points between COVID-19 positive and negative subjects (based on the average conditional minimal detectable change for Neuro-QoL anxiety and depression T-scores), assuming a United States COVID-19 positivity rate of 4%, power of 0.80, alpha of 0.05, and sample size of 820 participants. Patients were coded as having a worse than average NeuroQoL metric if their T-score was >55 (for anxiety, depression, fatigue, or sleep), or <45 (for cognition) based on data from reference populations (where mean T-score is set at 50 and the average minimal clinically significant difference in scores is 5) (Neuro-QoL, 2021).

Demographics, past medical history, stressors and new or worsened symptoms within the past month and NeuroQoL T-scores were compared between COVID-19 status groups using Chi-squared, Fisher’s Exact and Kruskal–Wallis non-parametric tests, as appropriate. Backwards step-wise, multivariable logistic regression models were constructed to predict worse than average Neuro-QoL scores (dichotomized at T-score >55 for anxiety, depression, fatigue or sleep and <45 for cognition) using the following covariates: age, race, ethnicity, sex, years of education, region of United States (U.S. Census Bureau Map of the United States Showing Census Divisions and Regions, 2021), population center (urban, suburban, and rural), COVID-19 status (negative, prolonged, positive but not prolonged), history of lung disease, history of depression/mood/thought disorder, and individual stressors within the last month (social isolation, financial insecurity, unemployment, food insecurity, homelessness, death of family member/friend, illness of family member/friend, fear of illness, new disability, education disruption, increased caregiver responsibilities, lack of access to childcare, political conflict with family/friends/colleagues, relationship issues with household, and domestic abuse/violence). This study was deemed IRB exempt per the NYU Langone Hospitals IRB. All analyses were conducted using IBM SPSS Statistics for Windows version 25 (IBM Corp., Armonk, NY, United States).

## Results

Of 1,000 responses, 999 were included in analysis and one duplicate was removed. Data were complete in 99.7% of responses. The average age was 45 years (range 18–84), 49% were male, and 77% were white. Respondents closely approximated United States census statistics for age, gender, race and region of United States, however, fewer Hispanics participated in this survey than are represented in the general United States population (Table 1). Overall, 76/999 (7.6%, 95% CI 6.0–9.4%) reported having COVID-19, either diagnosed by laboratory test (*N* = 46/76, 61%, 95% CI 49–72%) or by exposure to a known COVID-19 contact followed by typical symptoms (*N* = 30/76, 39%, 95% CI 28–51%). There were no statistically significant differences in demographics between patients who were diagnosed by laboratory or symptom-based criteria (Supplementary Table 2), however, those with symptom-based diagnoses tended to have COVID earlier in the pandemic, were more often from urban areas and were more often from the Northeast. This may reflect the limited COVID-19 testing that was available during the beginning of the pandemic in New York City region. No respondents were hospitalized for COVID-19. Of those with reported COVID-19, 19/76 (25%, 95% CI 16–36%) reported prolonged COVID-19 symptoms lasting a median of 4 months (range 1–13). Of these 19, 13 (68%) had laboratory confirmation and 6 (32%) had a COVID-19 exposure followed by typical symptoms. At least one stressor was identified in 676/999 (68%, 95% CI 65–71%) subjects within the last month and 648/999 (65%, 95% CI 62–68%) reported at least one new/worsened symptom since the onset of the pandemic. Overall, worse than average Neuro-QOL scores for anxiety, depression, fatigue, cognition and sleep occurred in 335/999 (34%, 95% CI 31–37%), 220/999 (22%, 95% CI 19–25%), 164/999 (16%, 95% CI 14–19%), 313/999 (31%, 95% CI 29–34%), and 279/999 (28%, 95% CI 25–31%), respectively.

**FIGURE 1:**
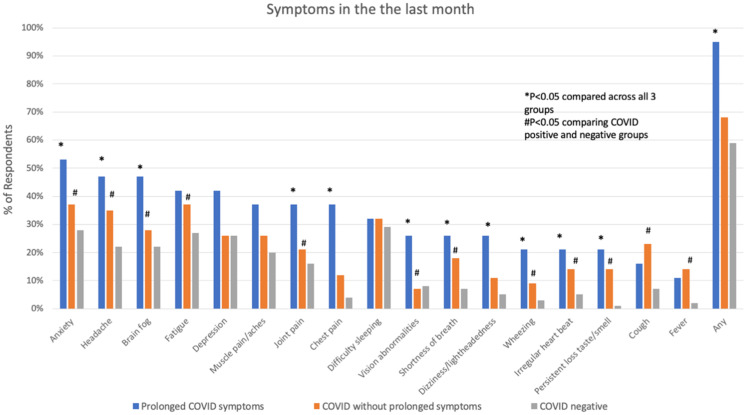
Symptoms in the month prior to interview in subjects with prolonged COVID-19 symptoms, COVID-19 without prolonged symptoms and COVID-19 negative subjects. *P < 0.05 comparing across all three groups; ^#^*P* < 0.05 comparing COVID positive to COVID negative.

**FIGURE 2:**
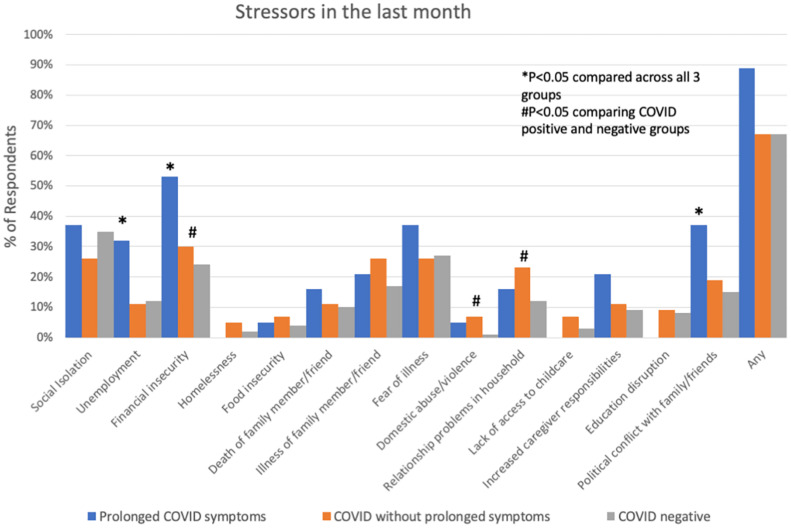
Socio-economic stressors in the month prior to interview in subjects with prolonged COVID-19 symptoms, COVID-19 without prolonged symptoms and COVID-19 negative subjects. *P < 0.05 comparing across all three groups; ^#^*P* < 0.05 comparing COVID positive to COVID negative.

Comparing respondents with a history of COVID-19 to those without, those with COVID were younger, more often Hispanic, and more often had a history of lung disease (Table 2). COVID-19 respondents had a significantly higher number of symptoms, specifically brain fog, headache, shortness of breath, cough, wheezing, chest pain, irregular heartbeat, fatigue, post-exertional malaise/brain fog, persistent loss of taste/smell, fever, dizziness/lightheadedness, and anxiety (Figure 1). These symptoms were more likely to interfere with work or household responsibilities compared to COVID negative respondents. COVID-19 respondents also had significantly higher rates of financial insecurity, domestic violence/abuse, or relationship problems with members of their household, though the total number of stressors experienced did not differ between COVID positive and negative groups (Figure 2).

**TABLE 2:** Characteristics of survey participants (*N* = 999).

	Prolonged COVID-19 symptoms^#^ *N* = 19	COVID-19 positive^∧^ without prolonged symptoms *N* = 57	COVID-19 negative *N* = 923	P*	*P*^¶^
**Demographics**					
Age, median (IQR)	32 (22–51)	44 (28–56)	45 (31–60)	**0.010**	**0.013**
Sex (male), *N* (%)	6 (32%)	37 (65%)	447 (48%)	**0.017**	0.172
Race, *N* (%)				**0.039**	0.283
White	13 (68%)	50 (88%)	702 (76%)		
Black	3 (16%)	7 (12%)	120 (13%)		
Asian	0	0	68 (7%)		
Native American/Alaskan Native	0	0	4 (0.4%)		
Pacific Islander/Native Hawaiian	0	0	2 (0.2%)		
Other	3 (16%)	0	23 (3%)		
Unknown/prefer not to answer	0	0	4 (0.4%)		
Ethnicity, *N* (%)				**<0.001**	**0.023**
Hispanic	5 (26%)	3 (5%)	39 (4%)		
Non-Hispanic	14 (74%)	54 (95%)	868 (96%)		
Years of education, median (IQR)	15 (14–16)	16 (14–18)	16 (14–17)	0.265	0.928
Region of United States^∗∗^, *N* (%)				0.276	0.102
North East	4 (21%)	10 (18%)	183 (20%)		
Mid-West	2 (11%)	13 (23%)	194 (21%)		
South	11 (58%)	29 (51%)	373 (40%)		
West	2 (11%)	5 (9%)	173 (19%)		
Population center, *N* (%)				0.310	0.179
Urban	10 (53%)	20 (35%)	263 (29%)		
Suburban	8 (42%)	30 (53%)	506 (55%)		
Rural	1 (5%)	7 (12%)	152 (17%)		
Time from COVID diagnosis to survey, median (IQR)	4 months (2–9 months)	2 months (<1–6 months)	–	0.255^Υ^	
**Past medical history, *N* (%)**					
None	13 (68%)	32 (56%)	532 (58%)	0.621	0.790
Hypertension	3 (16%)	14 (25%)	243 (26%)	0.565	0.499
Diabetes	1 (5%)	4 (7%)	100 (11%)	0.497	0.330
Coronary artery disease	1 (5%)	0	24 (3%)	0.351	1.00
Peripheral artery disease	1 (5%)	0	8 (1%)	0.101	0.511
Arrhythmia	1 (5%)	3 (5%)	26 (3%)	0.486	0.278
Lung disease (COPD/asthma)	2 (11%)	8 (14%)	50 (5%)	**0.021**	**0.019**
Cancer	1 (5%)	6 (11%)	44 (5%)	0.159	0.101
Venous thromboembolism	0	0	14 (2%)	0.557	0.617
Chronic liver disease	0	0	4 (0.4%)	0.848	1.00
Chronic kidney disease	1 (5%)	1 (2%)	11 (1%)	0.287	0.260
Anemia	3 (16%)	5 (9%)	61 (7%)	0.251	0.233
**Neurological/psychiatric history, *N* (%)**					
None	6 (32%)	33 (58%)	535 (58%)	0.070	0.260
Stroke	1 (5%)	1 (2%)	8 (1%)	0.137	0.173
Head trauma	0	1 (2%)	23 (3%)	0.740	1.00
Seizure/epilepsy	0	1 (2%)	11 (1%)	0.828	1.00
Dementia	0	0	0	—	—
Fibromyalgia	2 (11%)	1 (2%)	30 (3%)	0.170	0.734
Chronic fatigue syndrome	0	0	0	—	—
Depression	11 (58%)	17 (30%)	252 (27%)	**0.013**	0.075
Anxiety	8 (42%)	18 (32%)	283 (31%)	0.562	0.520
Other mood disorder (e.g., bipolar)	5 (26%)	1 (2%)	47 (5%)	**<0.001**	0.284
Thought disorder	1 (5%)	0	4 (0.4%)	**0.011**	0.327
**Stressors in the last month, *N* (%)**					
Total number, median (IQR)	3 (1–5)	2 (0–4)	1 (0–3)	0.083	0.076
None	4 (21%)	19 (33%)	306 (33%)	0.538	0.606
Social isolation	7 (37%)	15 (26%)	323 (35%)	0.400	0.287
Unemployment	6 (32%)	6 (11%)	110 (12%)	**0.032**	0.322
Financial insecurity	10 (53%)	17 (30%)	225 (24%)	**0.014**	**0.031**
Homelessness	0	54 (5%)	15 (2%)	0.112	0.143
Food insecurity	1 (5%)	4 (7%)	39 (4%)	0.598	0.336
Death of family member/friend	3 (16%)	6 (11%)	89 (10%)	0.660	0.546
Illness of family member/friend	4 (21%)	15 (26%)	160 (17%)	0.215	0.118
Fear of illness	7 (37%)	15 (26%)	252 (27%)	0.641	0.757
Domestic abuse/violence	1 (5%)	4 (7%)	12 (1%)	**0.003**	**0.007**
Relationship problems in household	3 (16%)	13 (23%)	108 (12%)	**0.043**	**0.028**
New disability	0	0	0	—	—
Lack of access to childcare	0	4 (7%)	24 (3%)	0.111	0.158
Increased caregiver responsibilities	4 (21%)	6 (11%)	84 (9%)	0.201	0.244
Education disruption	0	5 (9%)	72 (8%)	0.430	1.00
Political conflict with family/friends	7 (37%)	11 (19%)	140 (15%)	**0.028**	0.070
**Qualitative Symptoms in the last month, *N* (%)**					
Total number, median (IQR)	6 (2–9)	2 (0–7)	1 (0–4)	**<0.001**	**<0.001**
None	1 (5%)	17 (32%)	371 (41%)	**0.004**	**0.009**
Brain fog, difficulty concentrating, forgetfulness	9 (47%)	16 (28%)	205 (22%)	**0.023**	**0.033**
Post-exertional brain fog	8 (42%)	6 (11%)	57 (6%)	**<0.001**	**<0.001**
Headache	9 (47%)	20 (35%)	201 (22%)	**0.003**	**0.001**
Cough	3 (16%)	13 (23%)	64 (7%)	**<0.001**	**<0.001**
Vision abnormalities	5 (26%)	4 (7%)	69 (8%)	**0.010**	0.180
Shortness of breath	5 (26%)	10 (18%)	62 (7%)	**<0.001**	**<0.001**
Wheezing	4 (21%)	5 (9%)	25 (3%)	**<0.001**	**<0.001**
Irregular heart beat	4 (21%)	8 (14%)	49 (5%)	**0.001**	**0.001**
Chest pain	7 (37%)	7 (12%)	34 (4%)	**<0.001**	**<0.001**
Fatigue	8 (42%)	21 (37%)	246 (27%)	0.088	**0.031**
Post-exertional malaise/fatigue	5 (26%)	8 (14%)	55 (6%)	**<0.001**	**0.001**
Joint pain	7 (37%)	12 (21%)	150 (16%)	**0.042**	0.056
Muscle pain/aches	7 (37%)	15 (26%)	182 (20%)	0.098	0.055
Difficulty sleeping	6 (32%)	18 (32%)	269 (29%)	0.904	0.654
Persistent loss taste/smell	4 (21%)	8 (14%)	11 (1%)	**<0.001**	**<0.001**
Fever	2 (11%)	8 (14%)	16 (2%)	**<0.001**	**<0.001**
Dizziness/lightheadedness	5 (26%)	6 (11%)	49 (5%)	**<0.001**	**0.004**
Anxiety	10 (53%)	21 (37%)	258 (28%)	**0.025**	**0.018**
Depression	8 (42%)	15 (26%)	243 (26%)	0.305	0.456
**Impact due to symptoms, *N* (%)**					
No limitations on activities	1 (5%)	24 (42%)	468 (51%)	**<0.001**	**0.003**
Interfering with work	10 (53%)	21 (37%)	163 (18%)	**<0.001**	**<0.001**
Interfering with household responsibilities	13 (68%)	21 (37%)	239 (26%)	**<0.001**	**<0.001**
Limiting leisure activities	12 (63%)	21 (37%)	229 (63%)	**<0.001**	**<0.001**

^#^Symptoms lasting ≥ 1 month in COVID-19 positive patients; 12/17 (71%) had laboratory confirmed SARS-CoV-2 infection. ^COVID-19 positive = laboratory confirmed or exposure to known COVID-19 positive person and subsequent symptoms consistent with COVID-19. *Compares COVID-19 positive with prolonged symptoms (*N* = 17), COVID-19 positive without prolonged symptoms (*N* = 57) and COVID-19 negative (*N* = 906) using Kruskal–Wallis test for continuous variables and Chi-Squared test for binary and categorical variables. ^¶^Compares COVID-19 positive and negative groups Mann–Whitney *U*/Wilcoxon rank sum. ^Υ^Compares COVID-19 subjects with and without prolonged symptoms. **North East = Maine, New Hampshire, Vermont, Massachusetts, Connecticut, Rhode Island, New York, New Jersey, Pennsylvania; South = Maryland, Delaware, Virginia, West Virginia, Kentucky, Tennessee, North Carolina, South Carolina, Georgia, Mississippi, Alabama, Florida, Arkansas, Louisiana, Texas, Oklahoma; Midwest = Ohio, Michigan, Indiana, Illinois, Wisconsin, Minnesota, Iowa, Missouri, Kansas, Nebraska, North Dakota, South Dakota; West = Alaska, Hawaii, Montana, Wyoming, Colorado, New Mexico, Arizona, Utah, Idaho, Nevada, California, Oregon, Washington. Bold indicates *P* < 0.05.

**FIGURE 3:**
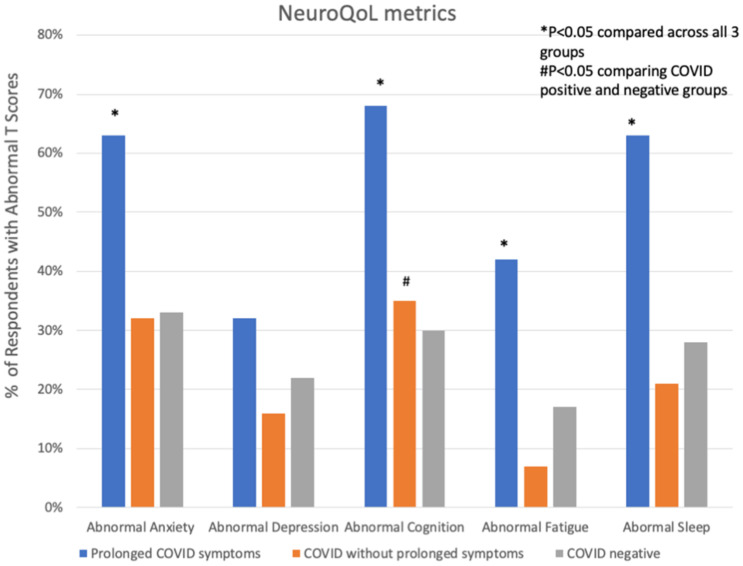
Abnormal NeuroQoL T-scores in subjects with prolonged COVID-19 symptoms, COVID-19 without prolonged symptoms and COVID-19 negative subjects. Abnormal T-scores were defined as: T-score > 55 for anxiety, depression, fatigue, or sleep and <45 for cognition. *P < 0.05 comparing across all three groups; ^#^*P* < 0.05 comparing COVID positive to COVID negative.

Participants with prolonged COVID-19 symptoms were more often younger, female, Hispanic, and had a history of depression, mood or thought disorder (Table 2). Stressors including unemployment, financial insecurity and political conflict were also more common in this group (Figure 2). The most common symptoms in the prolonged COVID-19 group were anxiety (53%), brain fog/difficulty concentrating/forgetfulness (47%), and headache (47%). This group experienced a greater number of symptoms in the prior month and were more disabled by these symptoms (symptoms interfered with work, household, or leisure activities) compared to other non-prolonged and COVID-19 negative respondents (Figure 3).

In univariate analyses, COVID-19 positive subjects (including those with and without prolonged symptoms) had significantly worse subjective measures of cognitive function than COVID-19 negative respondents, though NeuroQoL measures of fatigue, anxiety, depression and sleep symptoms did not differ between groups. Those with prolonged COVID-19 had significantly worse NeuroQoL T-scores and higher rates of worse than average symptoms of anxiety, cognition, fatigue and sleep than other groups (Table 3). After adjusting for demographic, past medical history and stressor covariates in multivariable logistic regression analysis, COVID-19 status (prolonged vs. not-prolonged vs. negative) was independently associated with worse Neuro-QoL cognitive dysfunction scores (adjusted OR 1.52, 95% CI 1.01–2.28, *P* = 0.047, Table 4), but there were no significant differences in quantitative measures of anxiety, depression, fatigue, or sleep. The most consistent factors significantly associated with worse NeuroQoL metrics across a variety of domains were: younger age, female gender, history of depression, social isolation, and relationship problems with members of the household (Table 4).

**TABLE 3:** Neuro-QoL T-scores by COVID-status (*N* = 999).

Metric	Prolonged COVID-19 symptoms *N* = 19	COVID-19 without prolonged symptoms *N* = 57	COVID-19 negative *N* = 923	P*	*P*^¶^
Anxiety T-score, median (IQR) Anxiety T-score > 55, *N* (%)	56.8 (51.4–62.6) 12/19 (63%)	53.3 (45.1–56.8) 18/57 (32%)	51.4 (45.9–57.6) 305/923 (33%)	0.007	0.201
Depression T-score, median (IQR) Depression T-score > 55^∧^, *N* (%)	51.3 (46.8–56.7) 6/19 (32%)	46.8 (36.9–53.2) 9/57 (16%)	47.9 (43.1–53.6) 205/923 (22%)	0.113	0.834
Cognition T-score, median (IQR) Cognition T-score <45^∧^, *N* (%)	41.9 (38.9–48.3) 13/19 (68%)	47.1 (42.4–54.2) 20/57 (35%)	50.9 (43.9–59.0) 280/923 (30%)	<0.001	0.001
Fatigue T-score, median (IQR) Fatigue T-score > 55^∧^, *N* (%)	54.4 (48.4–57.6) 8/19 (42%)	43.8 (40.7–50.8) 4/57 (7%)	45.6 (39.5–52.3) 152/922 (17%)	0.004	0.370
Sleep T-score, median (IQR) Sleep T-score > 55^∧^, *N* (%)	58.0 (50.4–62.8) 12/19 (63%)	47.3 (42.8–53.1) 12/57 (21%)	48.9 (41.7–55.6) 255/922 (28%)	0.004	0.432

IQR = interquartile range. ^∧^T-scores > 50 indicate *worse* than average self-reported health for anxiety, depression, fatigue and sleep compared to a reference United States population. T-scores > 50 indicate *better* self-reported cognitive function compared to a reference United States population. *Krusal–Wallis non-parametric test of continuous variable T-scores across all three COVID-19 status categories (prolonged COVID-19, non-prolonged COVID-19, COVID-19 negative. ^¶^Mann–Whitney *U*/Wilcoxon rank sum comparing COVID-19 positive and negative groups.

**TABLE 4:** Multivariable logistic regression models predicting worse than average Neuro-QoL metrics among subjects with prolonged COVID-19 symptoms (*N* = 19), COVID-19 without prolonged symptoms (*N* = 57), and COVID-19 negative (*N* = 923) subjects.

Variable	Adjusted Odds Ratio (95% CI)	*P*
**Neuro-QoL anxiety scores worse than average (T-score > 55)**		
Age	0.96 (0.95–0.97)	<0.001
Sex (male)	0.71 (0.51–0.98)	0.034
Years of education	0.92 (0.86–0.98)	0.009
History of depression/mood/thought disorder	3.48 (2.49–4.88)	<0.001
Social isolation	1.79 (1.27–2.53)	0.001
Relationship problem with member of household	2.64 (1.63–4.27)	<0.001
Fear of illness	1.98 (1.38–2.84)	<0.001
**Neuro-QoL depression scores worse than average (T-score > 55)**		
Age	0.97 (0.96–0.98)	<0.001
History of depression/mood/thought disorder	4.81 (3.38–6.84)	<0.001
Social isolation	1.93 (1.35–2.67)	<0.001
Unemployment	1.75 (1.10–2.80)	0.012
Relationship problem with member of household	1.70 (1.07–2.72)	0.025
**Neuro-QoL fatigue scores worse than average (T-score > 55)**		
Age	0.98 (0.97–0.99)	0.002
Sex (male)	0.63 (0.43–0.93)	0.021
Years of education	0.89 (0.82–0.96)	0.003
History of depression/mood/thought disorder	3.35 (2.27–4.95)	<0.001
History of lung disease (asthma/COPD)	2.29 (1.16–4.50)	0.017
Social Isolation	1.69 (1.12–2.54)	0.012
Relationship problem with member of household	2.35 (1.44–3.85)	0.001
Fear of illness	1.63 (1.07–2.47)	0.022
Political conflict with family/friends/colleagues	1.61 (1.02–2.56)	0.043
**Neuro-QoL cognitive dysfunction scores worse than average (T-score <45)**		
Age	0.97 (0.96–0.98)	<0.001
Years of education	0.92 (0.86–0.98)	0.007
History of depression/mood/thought disorder	3.46 (2.49–4.79)	<0.001
Social Isolation	2.30 (1.66–3.19)	<0.001
Food insecurity	2.42 (1.15–5.07)	0.020
Illness of family member/friend	1.74 (1.18–2.57)	0.005
Political conflict with family/friends/colleagues	1.66 (1.10–2.51)	0.016
COVID-19 status (negative, positive/no prolonged symptoms, positive with prolonged symptoms)	1.52 (1.01–2.28)	0.047
**Neuro-QoL sleep scores worse than average (T-score > 55)**		
Age	0.98 (0.97–0.99)	<0.001
Sex (male)	0.60 (0.44–0.83)	0.002
Hispanic ethnicity	2.25 (1.14–4.56)	0.020
History of depression/mood/thought disorder	3.24 (2.33–4.50)	<0.001
Education disruption	2.09 (1.21–3.61)	0.008
Social Isolation	1.74 (1.24–2.43)	0.001
Death of family member/friend	1.70 (1.04–2.81)	0.036
Food insecurity	3.07 (1.49–6.32)	0.002
Political conflict with family/friends/colleagues	1.86 (1.23–2.80)	0.003

*Covariates included in backward stepwise logistic regression analysis: age, sex, race (white versus other), ethnicity, years of education, region of United States, population center (rural, suburban, urban), COVID-19 status (negative, prolonged, positive but not prolonged), history of lung disease, history of depression, mood or thought disorder, and stressors within the last month (social isolation, financial insecurity, unemployment, food insecurity, homelessness, death of family member/friend, illness of family member/friend, fear of illness, new disability, education disruption, increased caregiver responsibilities, lack of access to childcare, political conflict with family/friends/colleagues, relationship issues with household, domestic abuse/violence). Variables not shown were not significant and did not remain in the final model.*

**FIGURE 4:**
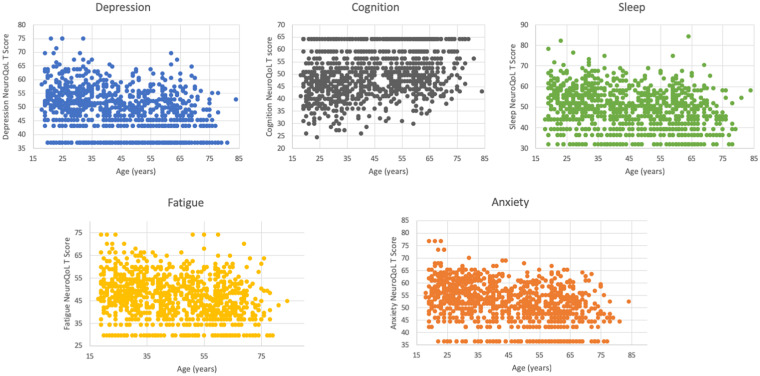
Distribution of Neuro-QoL T-scores by age. There is a suggestion of a bimodal peak in worse depression, cognitive and sleep score with peaks around ages 30–35 and again around ages 60–65.

We next explored the relationship of age with socio-economic stressors, symptoms and Neuro-QoL T-scores. Age was negatively correlated with both the number of symptoms (Spearman correlation coefficient −0.105, *P* = 0.001) and stressors (Spearman correlation coefficient −0.131, *P* < 0.001) experienced by subjects in the month prior to the survey. Notably, unemployment (Spearman correlation coefficient −0.146, *P* < 0.001) and financial insecurity (Spearman correlation coefficient −0.129, *P* < 0.001) were most strongly associated with younger age. Additionally, older respondents were less likely to report limitations in their routine activities due to symptoms (OR 0.98, 95% CI 0.97–0.99, *P* < 0.001). Overall, while age was inversely related to worse Neuro-QoL T-scores in multivariable analyses (Table 4), there was a suggestion of a bimodal distribution of worse T-scores for depression, cognition and sleep with peaks around ages 30 and again at 60–65 (Figure 4).

## Discussion

This study is the first, to our knowledge, to establish baseline quantitative measures of the prevalence of common symptoms of “long-hauler” syndrome – including cognitive dysfunction, fatigue, anxiety, depression, and sleep disorders – in a representative population of community-dwelling United States residents with and without a history of COVID-19. Furthermore, this is one of the first studies to examine the impact of psychosocial stressors on quantitative measures of subjective cognitive status, mood, and sleep. We found worse than average NeuroQoL scores occurred in ∼30% of subjects *without* COVID-19 across a range of domains. We further demonstrated that financial and social stressors, which may be exacerbated by the pandemic, predict worse NeuroQoL outcomes independent of COVID-19 status. However, a history of COVID-19, and particularly prolonged COVID-19, was associated with significantly worse subjective cognitive dysfunction scores, even after adjusting for baseline differences in demographics, past medical history and stressors. Indeed, most of the abnormalities in NeuroQoL metrics were driven by the subgroup of COVID-19 patients with protracted symptoms. Though there was an association of COVID-19 status with subjective cognitive dysfunction, other socio-demographic factors, including younger age, female gender, history of depression, social isolation and relationship problems with members of the household, were much stronger predictors of worse NeuroQoL metrics.

The relationship between stressors, heightened inflammatory response, mood disorders, cognitive abnormalities and neurodegenerative disease has been well established (Watt and Panksepp, 2009; Cunningham, 2011; Murray et al., 2011, 2012; Jack et al., 2018; Nation et al., 2019; Parnetti et al., 2019; Sweeney et al., 2019; Jones et al., 2020). Indeed, elevated IL-6 levels, which correlate with COVID-19 severity (Aziz et al., 2020; Frontera et al., 2020, 2021; Pairo-Castineira et al., 2021; Zhu et al., 2021), have been associated with depression and alterations in activity in the subgenual cingulate cortex (Drevets et al., 2008; Harrison et al., 2009). Other symptoms, including fatigue, malaise, myalgias, and joint pain, commonly referred to as “sickness behavior”, are thought to be triggered by proinflammatory cytokines (IL-1α, IL-1β, IL-6, and TNFα) generated as an innate immune response (Watt and Panksepp, 2009). The inflammatory response generated by pandemic-related stressors may represent the mechanistic underpinning of debilitating symptoms in patients that did not have COVID-19, though more data is needed to support this hypothesis. Among patients with a history of COVID-19, the inflammatory response or cytokine release syndrome associated with infection (Aziz et al., 2020; Leisman et al., 2020; Pairo-Castineira et al., 2021; Zhu et al., 2021) may synergize with a stressor-related inflammatory response to amplify and prolong post-viral symptoms.

Prolonged COVID-19 symptoms occurred in 25% of participants and were disabling, lasted months following diagnosis, and interfered with work and household responsibilities. The most common protracted symptoms reported in our study were anxiety, headache, “brain fog,” and fatigue, which have also been observed in other cohorts (Carfi et al., 2020; Huang et al., 2021; Mahmud et al., 2021; Tenforde et al., 2021). During the timeframe of this survey there were 26,779,193 United States confirmed COVID-19 cases (U. S. Johns Hopkins Coronavirus Resource Center, 2021), representing 8.1% of the total United States population (U.S. Census Bureau and U.S. World Population Clock, 2021). Our study detected a 7.6% COVID-19 positivity rate, which closely approximates population-based prevalences. Extrapolating from the 25% rate of prolonged symptoms among COVID-19 subjects in our study, there could be as many as 6,694,798 people in the United States currently experiencing post-acute sequelae of COVID-19 or “long-hauler” syndrome. Other countries have also documented prolonged symptoms following SARS-CoV-2 infection. A study conducted in the United Kingdom identified higher rates of stroke, dementia, mood and anxiety disorders 6-months after COVID-19 diagnosis compared to contemporaneous patients diagnosed with a different respiratory tract infections (Taquet et al., 2021). Similarly, a Chinese study found that 6-months after hospital discharge for COVID-19, 63% of patients had fatigue or muscle weakness, 26% had sleep abnormalities and 23% had anxiety or depression (Huang et al., 2021).

While we initially hypothesized that NeuroQoL metrics would be worse among older respondents, in fact, we detected the opposite. Older participants had fewer post-COVID symptoms and were less likely to have limitations in their activities due to symptoms. Older participants also reported fewer stressors. It is possible that the synergistic relationship between viral and stressor-related inflammatory responses was more pronounced in younger respondents. Financial insecurity and unemployment issues were significantly more common in those with prolonged COVID symptoms and these stressors were also inversely correlated with age, perhaps because younger people are less established in their careers, or have less savings. We did, however, detect the suggestion of a bimodal peak, where NeuroQoL scores for depression, sleep and subjective cognitive function appeared to worsen around ages 60–65. The underlying mechanisms related to abnormal scores in these different age ranges may differ and further evaluation is merited.

Limitations of this study include the fact that people who complete online surveys may not be representative of the general United States population in unmeasurable ways. This may limit generalizability to the United States population as a whole. However, our respondent population did closely approximate easily measured United States demographic data, though Hispanics were underrepresented. Second, it is possible we underestimated the prevalence of prolonged COVID symptoms since some of the respondents may have been diagnosed with COVID close to the time of the survey and hence not accrued enough time to qualify for prolonged COVID symptoms. Though the median time from COVID diagnosis to the survey was not statistically different between patients with or without prolonged symptoms, the median time interval was twice as long for those with prolonged symptoms compared to those without (4 versus 2 months). Third, cognitive function and financial stressors may be even worse than we measured, since the ability to complete an online survey requires access to technology and computer competence. Despite this, 29% of respondents reported unemployment, financial insecurity, food insecurity, or homelessness within the month prior to completing the survey, indicating that this was not a rarified group of respondents. Fourth, patients hospitalized with more severe COVID-19 may have substantially different outcomes than this cohort of non-hospitalized community dwellers. Fifth, the number of patients with COVID-19 was relatively small, as was the number of respondents with prolonged-COVID symptoms. However, we powered our survey for an even smaller positivity rate and our data provides important epidemiological information regarding the prevalence of post-acute COVID symptoms. Sixth, because this was a survey we had to rely on self-reported COVID status. Methodologically, obtaining laboratory proof of SARS-CoV-2 infection from all respondents would not have been feasible and our definition of COVID status was the only pragmatic option to obtain data rapidly on a large scale during a pandemic. Because respondents with symptom-based diagnoses tended to be from urban areas, the Northeast and had COVID earlier in the pandemic, it is possible that this group represents the first wave in the New York City area before testing was widely available. Last, NeuroQoL metrics are subjective measures of self-reported health. Objective measures of cognitive dysfunction using formal neuropsychological testing are needed to identify domains of dysfunction, which would guide therapeutic intervention.

## Conclusion

Prolonged symptoms lasting a medina of 4 months occurred in 25% of COVID-19 positive participants. NeuroQoL cognitive dysfunction scores were significantly worse among COVID-19 positive subjects, even after accounting for demographic and stressor covariates. Fatigue, anxiety, depression, and sleep scores did not differ between COVID-19 positive and negative respondents. Major factors associated with worse NeuroQoL metrics across a variety of domains were younger age, female gender, history of depression, social isolation, and relationship problems with members of the household.

## Data Availability Statement

The data will be made available to investigators upon reasonable request to the corresponding author.

## Ethics Statement

The studies involving human participants were reviewed and approved by NYU IRB. Written informed consent for participation was not required for this study in accordance with the national legislation and the institutional requirements.

## Author Contributions

JF designed the study, analyzed the data, and drafted the manuscript. AL, KM, JL, DK, RH, SY, SM, TW, LB, and SG contributed to the conceptual design of the study, data interpretation, and critical revision of the manuscript. All authors approved the submitted version of the manuscript.

## Conflict of Interest

The authors declare that the research was conducted in the absence of any commercial or financial relationships that could be construed as a potential conflict of interest.

## Footnotes

1 https://www.prolific.co
