# Supplementary material for: Prevalence and Predictors of Prolonged Cognitive and Psychological Symptoms Following COVID-19 in the United States
Source: Front Aging Neurosci. 2021 Jul 19;13:690383. doi: 10.3389/fnagi.2021.690383 (PMC8326803; doi:10.3389/fnagi.2021.690383)
Supplement: Supplementary file 1 [file Table_1.DOCX]

**Supplemental Table 1. Survey entitled “Prevalence of Medical Conditions among Community Dwellers”**

**Introduction to Survey**

“We are conducting a research study about the frequency of certain medical conditions among U.S. residents to see how they may affect quality of life. You are eligible to take part in this study if you are ≥18 years of age and are a U.S. resident. In this study, you will be asked to complete a one-time survey online about your health. It will take about 10-15 minutes of your time. Your survey responses may be used and shared with others in connection with this study. The research team, including the Principal Investigator, study coordinators, and personnel responsible for the support or oversight of the study may use and share information in connection with this study. What are the risks and benefits of being in this study? Risks: You may experience frustration when completing surveys. Some questions may be of a sensitive nature, and you may therefore become upset as a result. If you are experiencing depression or thoughts of suicide, we encourage you to contact your healthcare provider or call the National Suicide Prevention Lifeline at 800-273-8255. There is also a risk of breach of confidentiality. To reduce this risk, we do not collect any identifiers from you. Your Prolific ID is collected solely for payment purposes and we will not collect any further personal identification information. Benefits: The reimbursement for this study is $9.60/hour. We anticipate that this survey will take 10-15 minutes to complete. Therefore, we estimate that you will be paid $1.60-$2.40 by Prolific.co.”

| **Question** | **Responses** |
| --- | --- |
| What is your age (years)? |  |
| What was your sex assigned at birth? | \| 1 \| Female \| \| --- \| --- \| \| 2 \| Male \| \| 3 \| Intersex \| |
| What is your race? | \| 1 \| American Indian or Alaska Native \| \| --- \| --- \| \| 2 \| Asian \| \| 3 \| Black or African-American \| \| 4 \| Native Hawaiian or other Pacific Islander \| \| 5 \| White \| \| 6 \| Other \| \| 7 \| Unknown or prefer not to answer \| |
| What is your ethnicity? | \| 1 \| Hispanic or Latino \| \| --- \| --- \| \| 2 \| Not Hispanic or Latino \| |
| How many years of education have you completed starting at first grade? For example, completing high school equals 12 years, completing college equals 16 years, etc.  Please only count years that were completed. |  |
| What state do you live in? |  |
| What type of area do you live in? | \| 1 \| Urban \| \| --- \| --- \| \| 2 \| Suburban \| \| 3 \| Rural \| |
| Please check the box next to any medical condition that you have (now or in the past) or for which you take medication | \| High blood pressure \| \| --- \| \| Diabetes \| \| Heart disease (coronary artery disease, heart attack) \| \| Arrhythmia (abnormal heart beat) \| \| Lung disease (e.g. COPD, asthma) \| \| Cancer \| \| Peripheral artery disease or peripheral vascular disease \| \| History of blood clots in legs or lungs \| \| Chronic liver disease \| \| Chronic kidney disease \| \| Anemia \| \| None \| |
| Please check the box next to any neurological/psychiatric condition that you have (now or in the past) or for which you take medication | \| Fibromyalgia \| \| --- \| \| Chronic fatigue syndrome \| \| Stroke \| \| Head trauma \| \| Seizure or epilepsy \| \| Dementia or memory loss \| \| Spinal cord injury \| \| Depression \| \| Anxiety \| \| Other mood disorder (e.g. bipolar) \| \| Thought disorder (e.g. schizophrenia) \| \| None \| |
| **Section Header: *Anxiety***  ***Please mark the frequency with which you experienced the below sentiments in the last 7 days.***  **In the past 7 days...** | |
| I felt uneasy | \| 1 \| Never \| \| --- \| --- \| \| 2 \| Rarely \| \| 3 \| Sometimes \| \| 4 \| Often \| \| 5 \| Always \| |
| I felt nervous | \| 1 \| Never \| \| --- \| --- \| \| 2 \| Rarely \| \| 3 \| Sometimes \| \| 4 \| Often \| \| 5 \| Always \| |
| Many situations made me worry | \| 1 \| Never \| \| --- \| --- \| \| 2 \| Rarely \| \| 3 \| Sometimes \| \| 4 \| Often \| \| 5 \| Always \| |
| My worries overwhelmed me | \| 1 \| Never \| \| --- \| --- \| \| 2 \| Rarely \| \| 3 \| Sometimes \| \| 4 \| Often \| \| 5 \| Always \| |
| I felt tense | \| 1 \| Never \| \| --- \| --- \| \| 2 \| Rarely \| \| 3 \| Sometimes \| \| 4 \| Often \| \| 5 \| Always \| |
| I had difficulty calming down | \| 1 \| Never \| \| --- \| --- \| \| 2 \| Rarely \| \| 3 \| Sometimes \| \| 4 \| Often \| \| 5 \| Always \| |
| I had sudden feelings of panic | \| 1 \| Never \| \| --- \| --- \| \| 2 \| Rarely \| \| 3 \| Sometimes \| \| 4 \| Often \| \| 5 \| Always \| |
| I felt nervous when my normal routine was disturbed | \| 1 \| Never \| \| --- \| --- \| \| 2 \| Rarely \| \| 3 \| Sometimes \| \| 4 \| Often \| \| 5 \| Always \| |
| **Section Header: *Depression***  ***Please mark the frequency with which you experienced the below sentiments in the last 7 days.***  **In the past 7 days...** | |
| I felt depressed | \| 1 \| Never \| \| --- \| --- \| \| 2 \| Rarely \| \| 3 \| Sometimes \| \| 4 \| Often \| \| 5 \| Always \| |
| I felt hopeless | \| 1 \| Never \| \| --- \| --- \| \| 2 \| Rarely \| \| 3 \| Sometimes \| \| 4 \| Often \| \| 5 \| Always \| |
| I felt that nothing could cheer me up | \| 1 \| Never \| \| --- \| --- \| \| 2 \| Rarely \| \| 3 \| Sometimes \| \| 4 \| Often \| \| 5 \| Always \| |
| I felt that my life was empty | \| 1 \| Never \| \| --- \| --- \| \| 2 \| Rarely \| \| 3 \| Sometimes \| \| 4 \| Often \| \| 5 \| Always \| |
| I felt worthless | \| 1 \| Never \| \| --- \| --- \| \| 2 \| Rarely \| \| 3 \| Sometimes \| \| 4 \| Often \| \| 5 \| Always \| |
| I felt unhappy | \| 1 \| Never \| \| --- \| --- \| \| 2 \| Rarely \| \| 3 \| Sometimes \| \| 4 \| Often \| \| 5 \| Always \| |
| I felt I had no reason for living | \| 1 \| Never \| \| --- \| --- \| \| 2 \| Rarely \| \| 3 \| Sometimes \| \| 4 \| Often \| \| 5 \| Always \| |
| I felt that nothing was interesting | \| 1 \| Never \| \| --- \| --- \| \| 2 \| Rarely \| \| 3 \| Sometimes \| \| 4 \| Often \| \| 5 \| Always \| |
| **Section Header: *Fatigue***  ***Please mark the frequency with which you experienced the below sentiments in the last 7 days.***  **In the past 7 days...** | |
| I felt exhausted | \| 1 \| Never \| \| --- \| --- \| \| 2 \| Rarely \| \| 3 \| Sometimes \| \| 4 \| Often \| \| 5 \| Always \| |
| I felt that I had no energy | \| 1 \| Never \| \| --- \| --- \| \| 2 \| Rarely \| \| 3 \| Sometimes \| \| 4 \| Often \| \| 5 \| Always \| |
| I felt fatigued | \| 1 \| Never \| \| --- \| --- \| \| 2 \| Rarely \| \| 3 \| Sometimes \| \| 4 \| Often \| \| 5 \| Always \| |
| I was too tired to do my household chores | \| 1 \| Never \| \| --- \| --- \| \| 2 \| Rarely \| \| 3 \| Sometimes \| \| 4 \| Often \| \| 5 \| Always \| |
| I was too tired to leave the house | \| 1 \| Never \| \| --- \| --- \| \| 2 \| Rarely \| \| 3 \| Sometimes \| \| 4 \| Often \| \| 5 \| Always \| |
| I was frustrated by being too tired to do the things I wanted to do | \| 1 \| Never \| \| --- \| --- \| \| 2 \| Rarely \| \| 3 \| Sometimes \| \| 4 \| Often \| \| 5 \| Always \| |
| I felt tired | \| 1 \| Never \| \| --- \| --- \| \| 2 \| Rarely \| \| 3 \| Sometimes \| \| 4 \| Often \| \| 5 \| Always \| |
| I had to limit my social activity because I was tired | \| 1 \| Never \| \| --- \| --- \| \| 2 \| Rarely \| \| 3 \| Sometimes \| \| 4 \| Often \| \| 5 \| Always \| |
| **Section Header: *Cognition***  ***Please mark the frequency with which you experienced the below sentiments in the last 7 days as well as the difficulties you've encountered with various activities.***  **In the past 7 days...** | |
| I had to read something several times to understand it | \| 5 \| Never \| \| --- \| --- \| \| 4 \| Rarely (once) \| \| 3 \| Sometimes (2-3 times) \| \| 2 \| Often (once a day) \| \| 1 \| Very often (several times a day) \| |
| My thinking was slow | \| 5 \| Never \| \| --- \| --- \| \| 4 \| Rarely (once) \| \| 3 \| Sometimes (2-3 times) \| \| 2 \| Often (once a day) \| \| 1 \| Very often (several times a day) \| |
| I had to work really hard to pay attention or I would make a mistake | \| 5 \| Never \| \| --- \| --- \| \| 4 \| Rarely (once) \| \| 3 \| Sometimes (2-3 times) \| \| 2 \| Often (once a day) \| \| 1 \| Very often (several times a day) \| |
| I had trouble concentrating | \| 5 \| Never \| \| --- \| --- \| \| 4 \| Rarely (once) \| \| 3 \| Sometimes (2-3 times) \| \| 2 \| Often (once a day) \| \| 1 \| Very often (several times a day) \| |
| Section Header: *How much difficulty do you currently have...*  Reading and following complex instructions (e.g., directions for a new medication) | \| 1 \| None \| \| --- \| --- \| \| 2 \| A little \| \| 3 \| Somewhat \| \| 4 \| A lot \| \| 5 \| Cannot do \| |
| Planning for and keeping appointments that are not part of my daily routine | \| 1 \| None \| \| --- \| --- \| \| 2 \| A little \| \| 3 \| Somewhat \| \| 4 \| A lot \| \| 5 \| Cannot do \| |
| Managing time to do most daily activities | \| 1 \| None \| \| --- \| --- \| \| 2 \| A little \| \| 3 \| Somewhat \| \| 4 \| A lot \| \| 5 \| Cannot do \| |
| Learning new tasks or instructions | \| 1 \| None \| \| --- \| --- \| \| 2 \| A little \| \| 3 \| Somewhat \| \| 4 \| A lot \| \| 5 \| Cannot do \| |
| **Section Header: *Sleep disturbance***  ***Please mark the frequency with which you experienced the below sentiments in the last 7 days***  **In the past 7 days...** | |
| I had to force myself to get up in the morning | \| 1 \| Never \| \| --- \| --- \| \| 2 \| Rarely \| \| 3 \| Sometimes \| \| 4 \| Often \| \| 5 \| Always \| |
| I had trouble stopping my thoughts at bedtime | \| 1 \| Never \| \| --- \| --- \| \| 2 \| Rarely \| \| 3 \| Sometimes \| \| 4 \| Often \| \| 5 \| Always \| |
| I was sleepy during the daytime | \| 1 \| Never \| \| --- \| --- \| \| 2 \| Rarely \| \| 3 \| Sometimes \| \| 4 \| Often \| \| 5 \| Always \| |
| I had trouble sleeping because of bad dreams | \| 1 \| Never \| \| --- \| --- \| \| 2 \| Rarely \| \| 3 \| Sometimes \| \| 4 \| Often \| \| 5 \| Always \| |
| I had trouble falling asleep | \| 1 \| Never \| \| --- \| --- \| \| 2 \| Rarely \| \| 3 \| Sometimes \| \| 4 \| Often \| \| 5 \| Always \| |
| Pain woke me up | \| 1 \| Never \| \| --- \| --- \| \| 2 \| Rarely \| \| 3 \| Sometimes \| \| 4 \| Often \| \| 5 \| Always \| |
| I avoided or cancelled activities with friends because I was tired from having a bad night's sleep | \| 1 \| Never \| \| --- \| --- \| \| 2 \| Rarely \| \| 3 \| Sometimes \| \| 4 \| Often \| \| 5 \| Always \| |
| I felt physically tense during the middle of the night or early morning hours | \| 1 \| Never \| \| --- \| --- \| \| 2 \| Rarely \| \| 3 \| Sometimes \| \| 4 \| Often \| \| 5 \| Always \| |
| **Section Header: *Have you experienced any of the following stressors since the onset of the COVID-19 pandemic in the U.S. (March 2020) and/or within the last month ? (check all that apply since the pandemic [first column] and in the past month [second column])*** | |
| Social isolation |  |
| Unemployment |  |
| Financial insecurity |  |
| Homelessness |  |
| Food insecurity/lack of food |  |
| Death of a family member/friend |  |
| Illness of a family member/friend |  |
| Domestic abuse/violence |  |
| Relationship problem with member(s) of your household |  |
| Personal illness |  |
| Fear of illness |  |
| New disability |  |
| Lack of access to childcare |  |
| Caregiver responsibilities |  |
| Education disruption (for yourself or a member of your household) |  |
| Political conflict with family/friends/colleagues |  |
| Other |  |
| None |  |
| What other stressors have you had since the start of the pandemic? |  |
| What other stressors have you had in the past month? |  |
| **Section Header: *Have you experienced any of the following new or worsened symptoms that developed since the onset of the COVID-19 pandemic in the U.S. (March 2020) and/or within the last month? Please answer whether or not you have been diagnosed with COVID-19 (check all that apply since the pandemic [first column] and in the past month [second column])*** | |
| "Brain fog" or difficulty concentrating or unusual forgetfulness |  |
| Headache |  |
| Cough |  |
| Vision abnormalities (for example seeing spots, stars, lines, flashing lights, zigzag lines, heat waves or tiny dots on a gray, white or black background sometimes referred to as "snow") |  |
| Shortness of Breath |  |
| Wheezing |  |
| Irregular heartbeat or racing heart |  |
| Chest pain |  |
| Fatigue |  |
| Joint pain |  |
| Muscle pain/aches |  |
| Difficulty sleeping |  |
| Persistent loss of taste/smell |  |
| Fever |  |
| Dizziness/lightheadedness |  |
| Anxiety |  |
| Depression/sadness |  |
| Post-exertional malaise or fatigue |  |
| Post-exertional brain fog, loss of concentration or memory problems |  |
| Other (specify) |  |
| None |  |
| Specify other symptoms since the start of the pandemic |  |
| Specify other symptoms in the past month |  |
| Have any of the above symptoms interfered (or are currently interfering) with your activities? (Check all that apply) | \| work \| \| --- \| \| household responsibilities \| \| leisure activities \| \| other \| \| no limitations on activities \| |
| Specify other activity limitations due to above symptoms | text |
| Have you ever been tested for COVID-19? | \| 1 \| Yes \| \| --- \| --- \| \| 2 \| No \| \| 3 \| Unknown \| |
| What was the result of the test? (If you have been tested more than once and any test was positive, please check "positive") | \| 1 \| Positive \| \| --- \| --- \| \| 2 \| Negative \| \| 3 \| Unknown result \| |
| Have you had COVID-19 diagnosed by either a laboratory test OR by symptoms (fever >99.5, new onset cough, shortness of breath, muscle pain, headache, sore throat or loss of taste or smell) following an exposure to a patient with documented COVID-19? (check all that apply) | \| 1 Symptoms of COVID-19 (fever >99.5, new onset cough, shortness of breath, muscle pain, headache, sore throat or loss of taste or smell) AND Exposure to COVID-19 positive person \| \| --- \| \| 2 Positive laboratory tests for COVID-19 (including PCR or molecular test by nasal or nasal pharyngeal swab, rapid test or antigen test, antibody test) \| \| 3 None of the above \| |
| How long ago were you diagnosed with COVID-19? | \| < 1 month \| \| --- \| \| 1-3 months \| \| 3-6 months \| \| 6-9 months \| \| 9-12 months \| \| >12 months \| |
| Were you ever hospitalized for COVID-19? | \| 1 \| Yes \| \| --- \| --- \| \| 2 \| No \| |
| Do you believe you have "post-COVID syndrome" also called "long hauler" syndrome or do you continue to have symptoms weeks or months after your initial diagnosis of COVID-19? | \| 1 \| Yes \| \| --- \| --- \| \| 2 \| No \| |
| If you believe you have "post-COVID" or "long-hauler" syndrome, for how many months following your initial COVID-19 diagnosis have you continued to have symptoms? |  |
