# Supplementary material for: Prevalence and Predictors of Prolonged Cognitive and Psychological Symptoms Following COVID-19 in the United States
Source: Front Aging Neurosci. 2021 Jul 19;13:690383. doi: 10.3389/fnagi.2021.690383 (PMC8326803; doi:10.3389/fnagi.2021.690383)
Supplement: Supplementary file 2 [file Table_2.DOCX]

**Supplemental Table 2. Demographic comparison of subjects with laboratory based (N=46) and symptom based (N=30) COVID-19 diagnoses**

|  | **Laboratory diagnosed COVID-19**  **(N=46)** | **Symptom based diagnosis of COVID-19**  **(N=30)** | **P** |
| --- | --- | --- | --- |
| **Demographics** |  |  |  |
| Age, median (IQR) | 45 (26-55) | 38 (25-59) | 0.827 |
| Sex (male), N (%) | 23/46 (50%) | 20/30 (67%) | 0.152 |
| Race, N (%)  White  Black  Asian  Native American/Alaskan Native  Pacific Islander/Native Hawaiian  Other  Unknown/prefer not to answer | 38/46 (83%)  7/46 (15%)  -  -  -  1/46 (2%)  - | 25/30 (83%)  3/30 (10%)  -  -  -  230 (7%)  - | 0.521 |
| Ethnicity, N (%)  Hispanic  Non-Hispanic | 5/46 (11%)  41/46 (89%) | 3/30 (10%)  27/30 (90%) | 0.904 |
| Years of education, median (IQR) | 16 (14-17) | 16 (14-18) | 0.686 |
| Region of U.S^**^, N (%)  North East  Mid-West  South  West | 7/46 (15%)  13/46 (28%)  23/46 (50%)  3/46 (7%) | 7/30 (23%)  2/30 (7%)  17/30 (57%)  4/30 (13%) | 0.111 |
| Population center, N (%)  Urban  Suburban  Rural | 15/46 (33%)  26/46 (57%)  5/46 (11%) | 15/30 (50%)  12/30 (40%)  3/30 (10%) | 0.302 |
| Time from COVID diagnosis to survey, median (IQR) | 2 months  (1-6 months) | 4 months  (1-9 months) | 0.124 |
